# Cambridge Neoadjuvant Cancer of the Prostate (CANCAP03): A Window Study into the Effects of Olaparib ± Degarelix in Primary Prostate Cancer

**DOI:** 10.1158/1078-0432.CCR-24-1304

**Published:** 2025-06-13

**Authors:** Harveer Dev, Mark Linch, Krishna Narahari, Toby Milne-Clark, Melissa Cheung, Anne Warren, Alopa Malaviya, Vincent Gnanapragasam, Tatiana Hernandez, Nicholas Bullock, Andrea Machin, Alimu Dayimu, Tamsin Robb, Elizabeth Cromwell, Alex Freeman, Elizabeth A Harrington, Niedzika Camacho, Silvia Glont, Massimo Squatrito, Asaf Rotem, Luiza Moore, Robert Hanson, Marc Dodd, Shubha Anand, Howard Kynaston, Greg Shaw, Nimish Shah, Simon Pacey

**Affiliations:** 1 Early Cancer Institute, University of Cambridge; 2 University College London Cancer Institute; 3 University Hospital of Wales, Cardiff & Urology MDRG, Cardiff University; 4 Cambridge University Hospitals NHS Foundation Trust; 5 Department of. Urology, Cambridge University Hospitals NHS Foundation Trust; 6 Division of Urology, Department of Surgery, University of Cambridge; 7 START Barcelona-HM CIOCC Early Phase Program, Spain; 8 Clinical Trials Unit, Cancer Theme, University of Cambridge; 9 Translational Medicine, Oncology R&D, AstraZeneca, Cambridge CB2 0AA, UK; 10 Oncology Data Science, Oncology R&D, AstraZeneca, Cambridge, UK; 11 CMDL, Department of Oncology, University of Cambridge; 12 University College London Hospitals NHS Trust; 13 Department of Oncology, Clinical School, University of Cambridge

**Keywords:** Prostate Cancer, Prostatectomy, PARP inhibitor, olaparib, degarelix, window

## Abstract

**Purpose:**

Investigate combined PARP and androgen inhibition in primary prostate cancer. Aim: to understand the biological mechanism(s) underlying clinical efficacy, especially in the absence of mutations in homologous recombination (HR) repair pathways.

**Patients and Methods:**

Primary objective - measure PARP inhibition, secondary objectives - assess safety and feasibility. Participants received olaparib for two-weeks before prostatectomy, and randomly assigned (1:1) to degarelix or not. We analysed diagnostic biopsy and radical prostatectomy samples for PARylated protein expression using immunohistochemistry. Exploratory analyses included tumour gene sequencing, mutation analysis and RNA sequencing using both bulk and single-cell RNA sequencing performed on pre- and post-treatment tissue.

**Results:**

PARylated protein expression was significantly reduced in both cohorts, with no drug-related delays in radical prostatectomy. Gene set enrichment analysis identified distinct treatment response signatures related to olaparib in both cohorts, and showed downregulation of androgen response genes following olaparib + degarelix treatment.

Transcript profiling revealed an upregulation of the p53 hallmark, more pronounced with the combination treatment. Canonical cell cycle progression hallmarks including E2F targets and the G2M checkpoint, were suppressed across all cases, correlating with a HR deficient transcriptional signature. Single-nuclear RNA sequencing indicated a greater increase in inflammatory response pathway activity within tumour epithelia following combination treatment.

**Conclusions:**

Transcriptomic analysis identified common hallmark alterations reflecting the combined impact of PARPi and androgen blockade on cell cycle progression. We observed a shared phenotypic response to combination therapy across prostate cancers without known HR repair gene alterations. This suggests alternative mechanisms rather than anti-androgen induced HR deficiency.

## Introduction

Worldwide prostate cancer remains the second leading cause of male cancer related deaths (396 000 in 2022)^1^ Novel therapies have improved overall survival rates most notably by intensifying the earliest lines of therapy^2,3^). However, once prostate cancer has progressed further improvements are still needed

Cancer cells with deficient homologous recombination repair (HRR) rely on alternative DNA repair pathways which are blocked by Poly (ADP-ribose) polymerase (PARP) inhibitors, leading to cell death through synthetic lethality^4^. PAR-mediated chromatin relaxation is impeded and stable PARP-DNA complexes are formed trapping PARP onto DNA^5^. This complex obstructs the progressing replication fork which collapses during S-phase, causing a single-ended double strand DNA break^6^. In prostate cancer, defects in DNA repair genes are often identified^7^. Repair of the PARP inhibitor induced break requires HRR; ergo, cancer cells which are HR defective (e.g. harbouring an oncogenic *BRCA1* or *BRCA2* variant) accumulate DNA damage eventually leading to cell death and are thus sensitive to PARP inhibition^5,8,9^.

Phase III trials, that recruited men whose prostate cancer harboured HRR gene defects, confirmed the activity of single agent PARP inhibitors e.g. olaparib^10^ and rucaparib^11^. Additional pre-clinical evidence has linked AR and PARP pathways in two ways: PARP1 supports AR dependent transcription^12,13^ and, AR signalling inhibition downregulates HRR gene expression leading to an induced HRR-deficient phenotype ^13,14^.Various androgen receptor signalling inhibitors (ARSI)/PARP inhibitor combinations were explored in PROpel, MAGNITUDE and TALAPRO-2 clinical trials, in the first line setting for men with mCRPC^15–17^. Progression free survival was improved for men with mCRPC and altered HRR as well in those men whose tumours had no apparent HRR gene defects in the PROpel^15^ and TALAPRO-2 studies^17^. These data have raised the question as to whether prostate cancer patients lacking HRR gene defects should be treated with PARPi/ ARSI combination therapy. Beije and colleagues^18^ summarised the discussion, concluding that further studies were urgently required to better understand the mechanisms of action for PARPi/ ARSI combinations.

Neoadjuvant “window of opportunity” trials support drug development, for example by enabling the analysis of human tissue samples before and after dosing, or testing for signals of clinical activity. CANCAP03 involved the administration of PARP inhibitor ± Androgen deprivation for a short duration. Olaparib, a potent inhibitor of PARP enzymes^19^ was used and Androgen deprivation was achieved using degarelix (a selective gonadotrophin releasing-hormone (GnRH) antagonist chosen due to the rapid, reproducible, suppression of testosterone within hours/ days of dosing^20^. PARP inhibition was assessed by measuring changes in PARylated protein expression^21^ as a biomarker. PARP inhibition reduces levels of poly (ADP-Ribose) polymer “PAR”^22^ and this has been analysed as a biomarker of PARP inhibition in human clinical trials^23,24^. In addition we present the results from correlative studies that were planned to study the molecular biological consequences of PARP inhibitor ± androgen deprivation.

## Materials and Methods

The primary objective was to assess the degree of PARP inhibition in prostate tumour cells following treatment with olaparib (± degarelix). Secondary objectives were to assess: the feasibility, safety and tolerability of a short course of olaparib (± degarelix) given prior to radical prostatectomy; preliminary evidence of tumour response e.g. pathological changes and PSA levels, following treatment with olaparib (±degarelix). Exploratory objectives included assessing changes in relevant biological pathways following olaparib treatment (± degarelix) and to investigate the association of DNA repair defects with sensitivity to olaparib treatment (± degarelix).

### Participants

CANCAP03 was an open-label, multi-centre, window study of olaparib ± degarelix given in the two weeks prior to radical prostatectomy (ClinicalTrials.gov: NCT02324998). Participants were men aged ≥ 18 years old (recruited between April 2017 and July 2018) who gave written, informed consent. They were ECOG 0-1 and medically fit for radical prostatectomy. Either diagnostic biopsy tissue was available, or a repeat baseline biopsy was mandated. Participants had either high risk (≥ 1 of Stage T2c-3a, or PSA > 20ng/mL or Gleason score ≥ 8) or intermediate risk (≥ 2 of Stage T2 (any), PSA > 10ng/mL, Gleason ≥ 7) of prostate cancer recurrence. The study followed the principles within the Declaration of Helsinki and was approved by The South Central - Berkshire B Research Ethics Committee (IRAS 166367) and MHRA (EudraCT 2014-004417-86), extended follow up data were collected under CANCAP-Translational, approved by North East - Tyne & Wear South Research Ethics Committee (IRAS 287622). Cambridge University Hospitals NHS Foundation Trust and the University of Cambridge sponsored both trials. The study received funding from AstraZeneca and Ferring, with olaparib provided free of charge by AstraZeneca.

### Treatment

Participants were randomly allocated (1:1 ratio) and received olaparib alone or olaparib plus degarelix. Olaparib, 300mg twice daily, oral was given on days 1-15 and degarelix 240mg once, subcutaneous, on day 1. Olaparib dosing continued until the morning of surgery.

### Procedures

Screening occurred over a maximum 28-day period, except ECG, safety and research bloods (within 14 days) before day 1. Baseline, pre-dose, diagnostic tumour biopsy samples were collected (formalin fixed paraffin-embedded, FFPE), with safety bloods as well as PSA during screening. Participants were reviewed on day 8 and day 15 (radical prostatectomy, RP). Post dose tumour samples (FFPE and frozen) were taken during RP on Day 15 using established methods^25^ and a sample to measure PSA. Six-weeks post-prostatectomy PSA measurement was repeated and follow up was completed, unless ongoing adverse reactions required monitoring to resolution. Compliance was assessed by participant diary and returned tablet count. Adverse events (AEs) were recorded from the time participant’s provided informed consent was given until the end of participation in the trial using CTCAE version 4.03.

### Immunohistochemistry

Haematoxylin and eosin–stained sections from existing diagnostic FFPE prostate needle core biopsies and the RP specimens were reviewed. Both specimen types were processed according to standard methodology^26^ The study pathologist reviewed the stained sections and identified and outlined the index tumour nodule in the RP sections. A representative area was selected for analysis by immunohistochemistry. An area in the biopsies most representative of the RP tumour nodule was also selected for analysis.

PARP inhibition reduces levels of poly (ADP-Ribose) polymer “PAR”^22^ and used as a surrogate biomarker of PARP inhibition in clinical trials^27^. Immunostaining with mouse monoclonal [10H] to poly (ADP-Ribose) polymer “PAR” (ABCAM, ab14459) was performed on 3.5μm thick FFPE sections using an automated immunostainer (Bond-III system, Leica Biosystems). The optimal working dilution of the primary antibody was 1/400 and the antigen retrieval was performed on board using the combination of heat and Bond Epitope Retrieval 1 solution for 10 minutes. The ADP-Ribose was detected and visualized using the Bond Polymer Refine Detection Kit (Leica, DS9800). Human tonsil tissue was used as positive control, while the primary antibody was removed as a negative control.

The immunohistochemistry was assessed independently by two pathologists (specialising in urological pathology), blinded to the clinical outcome data. A semi-quantitative manual score (0-300) was derived by a visual assessment of the intensity of the nuclear staining, categorised as absent, weak, moderate, strong (0,1,2,3 respectively) and multiplying this by the percentage of tumour cells stained. Where there was a difference in the score between the two assessors, a consensus score was agreed on review.

### Statistical analysis

The primary outcome was to measure the degree of PARP inhibition comparing baseline and post-treatment values, measured by immunohistochemistry (IHC).

The secondary outcome measures were the incidence and severity of adverse events caused by treatment, the number of participants who underwent radical prostatectomy on schedule, and tumour response (determined by pathological review and change in PSA from baseline to end of treatment).

Because of the exploratory nature of this study, no formal sample size calculation was performed. A total of 20 evaluable participants (10 in each group) were planned and this is compatible with detecting a Cohen’s D ≥0.8 with a 20% significance level (one-sided) and 80% power.

The safety population comprised all participants enrolled, who received at least one dose of study drug(s). Participants were evaluable unless there was: technical failure to measure PARylated protein by IHC on the paired tissue samples, or non-completion of planned investigational medical product (IMP) course.

The difference between baseline and post-treatment measurements were compared with Wilcoxon Signed Rank Test, and Mann Whitney U Test was applied to test the difference of measurements between treatment group. Significance was defined by a p-value threshold of 0.05 with a two-sided test. The proportion of participants that underwent radical prostatectomy on the planned date and reason(s) for any delay were noted. Statistical analyses were performed using R (version 4.1.0). Remaining statistical analyses were descriptive including graphical distributions of relative changes (%) in tumour PARylated protein stain from baseline.

### Targeted sequencing and mutation analysis

Representative tumour tissue was taken from the FFPE tissue RP blocks, from areas outlined by the study pathologist on the corresponding Haematoxylin and Eosin–stained sections. Using clean disposal skin punches, 2mm diameter punches were taken from the marked areas. Between one and six tumour regions were pooled to ensure sufficient yield for downstream analyses (median = 3). DNA was extracted from the FFPE specimen using the Qiagen DNA extraction kit (QIAamp DNA FFPE Tissue kit). DNA was also extracted from normal prostate tissue (n=17), and/or matched buffy coat (n=17, using the QIAmp DNA Blood Mini Kit) as a normal control. Targeted sequencing libraries were prepared using a custom 350 gene panel (Total Size: 1.46 Mb) from TWIST Biosciences using the manufacturer’s protocol and were sequenced on an Illumina NextSeq 2000. All exons of 350 genes and flanking sequences (+/- 20bp) are targeted with this panel. (See Supplementary table 1 for list of genes). Sequencing data was analysed using an in-house bioinformatics pipeline, aligning to hg38, which utilizes the following main algorithms: SNVs/INDELS identified following GATK4/MuTect2 best practices from Broad Institute (RRID:SCR_001876).

The minimum coverage to call variants is 100x, and the targeted coverage is 500x. The limit of detection of the assay is 3% variant allele frequency. The algorithm may not detect complex deletions, duplications, and large genomic rearrangements. All known benign variants and SNPs present at >1% frequency in population databases (e.g. GnomAD, ExAC) were filtered out. All called variants were visually inspected in Integrative Genomics Viewer (IGV) and false positive calls were removed. Matched buffy coat and normal prostate tissue sequencing was used to distinguish between somatic and germline variants. Pathogenicity was assigned according to criteria recommended by AMP guidelines & using known databases e.g ClinVar (RRID:SCR_006169), OncoKB (RRID:SCR_014782) and MTB portal.

### RNA Sequencing

Pre-treatment biopsies and post-treatment RP, FFPE tissue samples were available for 19 participants, including 10 participants who received olapaprib monotherapy and 9 participants who received olapaprib and degarelix. The post treatment sample from participant 1013 had an inadequate tumour content for analysis. RNA was extracted by the Cambridge Molecular Diagnostic Laboratory using the ReliaPrep™ FFPE Total RNA (Promega).

Whole transcriptome library construction was conducted using the KAPA RNA HyperPrep RiboErase Kit HMR (Roche) on the automated Biomek i5 and i7 liquid handlers (Beckman coulter). RNA-seq libraries were quantified using the TapeStation instrument (Agilent) and then subjected to paired-end 150bp Illumina sequencing. This resulted in an average of 100M mapped reads per sample and approximately 35,000 detected genes per sample

Pre-processing of raw reads, alignment to hg38 and generation of transcript-level counts were performed using STAR and featureCounts^28^. Differential expression analysis was performed using DESeq2^29^. Gene set enrichment analyses were performed on gene lists, ranked by log2fold change with respect to pre-treatment control samples using the ClusterProfiler package^30^. Expression z-scores were calculated using the following formula: $Zscore\;=\frac{x_{post\;}-{\bar{x}}_{pre\;}}{\sigma_{pre\;}}$ where *x*_*post*_ is the post-treatment sample, ${\bar{x}}_{pre\;}$ is the mean of the pre-treatment samples and *σ*_*pre*_ is the standard deviation of the pre-treatment samples. Analysis of results from the DARANA trial (enzalutamide prior to RP, NCT03297385) were obtained from counts via the GEO DataSet GSE197780.

### Single-nuclear RNA Sequencing

Post-treatment radical prostatectomy tissue cores (from one participating site) were snap-frozen, with subsequent histopathological review to confirm tumour content. Tissue punch cores for six patients, 3 from each of olaparib and olaparib and degarelix cohorts, were processed for nuclear isolation and extraction, prior to single-nuclear RNA sequencing.

The cylindrical tissue punch cores were first cut laterally into two pieces, each ∼60mg. Each piece was then cut vertically, leading to four approximately equal sized parts per sample. Top left and bottom right pieces from opposite side were used for preparation of single nuclei suspension. Tissue was disintegrated using CP02 CryoPREP automated Dry Pulverizer (Covaris) and lysed with TST buffer (0.145M NaCl, 0.01M Tris-HCL pH 7.5, 0.001M CaCl_2_, 0.021M CaCl_2_, 0.03% Tween 20, 0.01% BSA, 1U/μl RNase inhibitor murine (New England Biolabs) and nuclease free water). Sample was resuspended in 2 ml TST buffer and incubated on ice for 10 minutes with pipette mixing at midpoint. Lysed nuclei suspension was filtered first by 100μm cell strainer, followed by 40μm cell strainer (Corning), and centrifuged at 500 g for 5 minutes at 4 °C. Nuclei washing was performed with PBS+0.04% BSA with 0.04U/μl RNase inhibitor murine, and stained with AO/PI (Nexcelom). Nuclei were counted using Cellometer Auto 2000 (Nexcelom), then adequate nuclei to target 10,000 nuclei were loaded into each channel of Chip M run on the 10x Chromium X controller instrument, following the manufacturer-supplied protocol 10x Genomics CG000416 Rev D for single cell 3’ high throughput assay. 10x libraries were constructed using the 10x supplied protocol, briefly: individual nuclei were trapped into droplets with a single bead, and poly A transcripts were barcoded during reverse transcription. Complementary DNA was amplified, and libraries were prepared for sequencing on NovaSeq 6000 using S4 flow cell (100k reads per cell)^31^

FASTQ files were processed using CellRanger pipeline (10X Genomics). Outputs from the CellRanger pipeline were analysed using the Seurat package (v4.9.9.9049, RRID:SCR_016341) in R (v4.2.0). Nuclei with less than 500 unique molecular identifiers (UMIs), less than 300 genes detected, and greater than 20% mitochondrial reads were discarded. Doublets were identified using the scDblFinder package (v1.12.0)^32^ (no doublet filter was applied). The number of nuclei retained after quality control filtering was 164,698. The data were normalised by SCTransform^33^ using the Gamma-Poisson generalized linear model in the glmGamPoi package (v1.10.2)^34^. Datasets from different treatment conditions were integrated using 3000 anchor features and the SCT normalisation method. Principal component analysis (PCA) was run using default parameters (50 principal components (PCs)). Graph-based clustering was performed on the integrated dataset using the FindNeighbours and FindClusters functions in Seurat, using the first 30 PCs for building the shared nearest neighbour graph and a resolution parameter of 0.5, resulting in 24 clusters. Differentially expressed markers in each cluster were identified using the FindAllMarkers function in Seurat. Clusters were annotated into cell types using established marker genes^35^), and clusters labelled with the same cell type were merged for downstream analysis. For differential expression analysis: pseudo-bulk samples were generated by aggregating counts for all nuclei within the same cell type. DE analysis between the olaparib and Combination treatment conditions was performed using quasi-likelihood (QL) methods from the edgeR package (v3.42.4, RRID:SCR_012802) provided within the pseudoBulkDGE function in the scran package (v1.28.2).

Gene set enrichment analysis was performed using clusterProfiler (v4.8.3), gene sets with false discovery rate (FDR) q-values < 0.05 were considered statistically significant. All subsequent analysis carried out as described in bulk RNA-seq methods.

## Results

### Demographics

**Figure 1:**
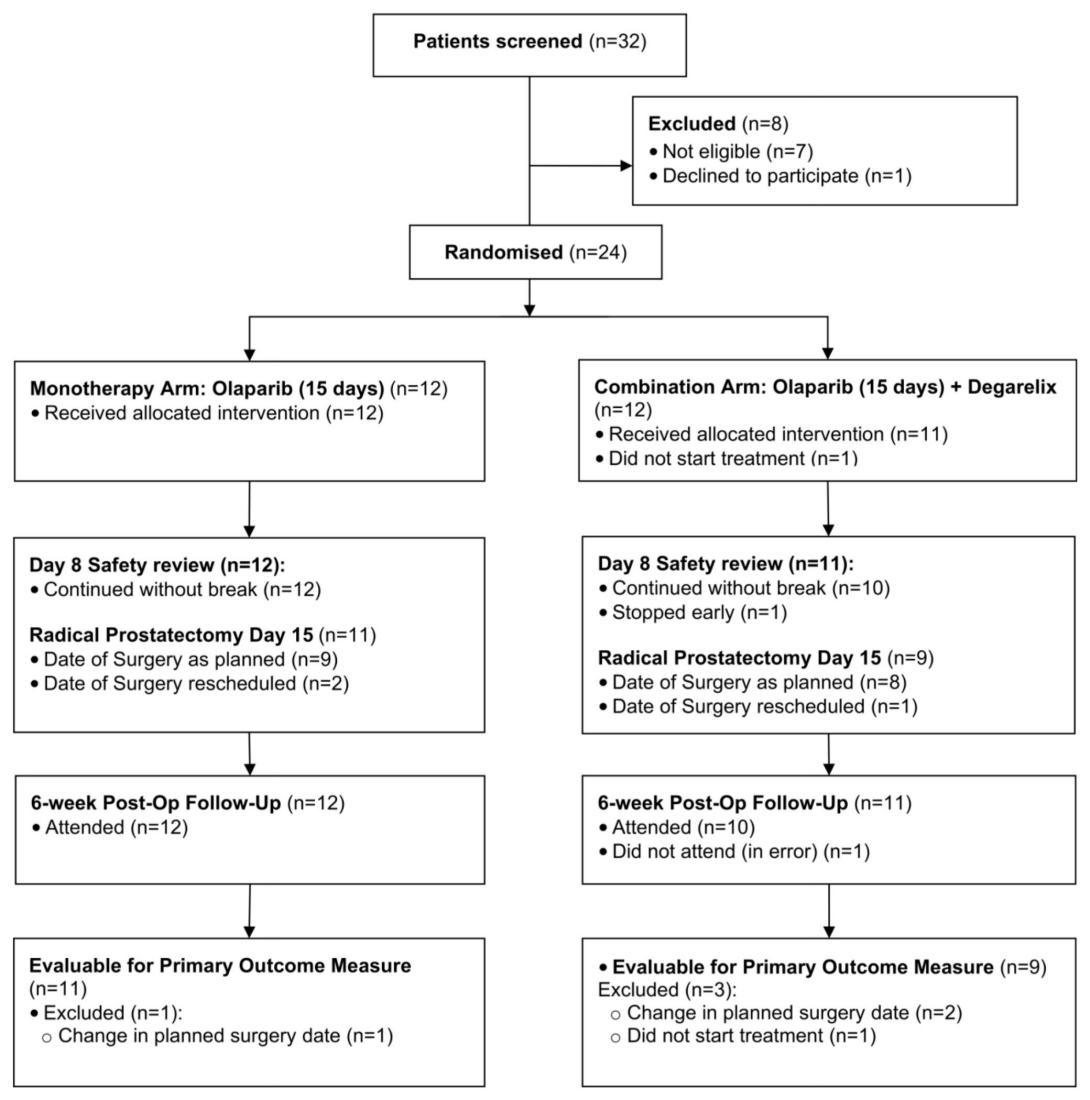
CONSORT diagram for CANCAP03 study.

A total of 24 participants (12 olaparib cohort, 12 olaparib + degarelix cohort) were recruited at three UK centres, with a study flow diagram outlined in Figure 1. The characteristics of evaluable study participants are summarised in Table 1 (for all randomised patients, see Supplementary Table 2). Participants in the olaparib cohort had a median age of 60 (range 47-71) years compared to 63 (49-69) in the olaparib + degarelix cohort. All participants had disease classified as D’Amico intermediate or high-risk of recurrence. Despite randomisation, with a small cohort size there was an apparent imbalance between Grade Groups between the two groups.

### Immunohistochemistry

Paired, pre and post treatment, samples were available for twenty participants who were evaluable for primary endpoint analysis (olaparib n=11 and combination n=9). Independent blinded pathology review occurred in all cases; discrepancies (3/20) were minor and resolved by mutual agreement on review. The date of prostatectomy was altered for three participants out of the 23 who received study medication, which affected dosing such that they were not evaluable for the primary endpoint analysis.

**Figure 2:**
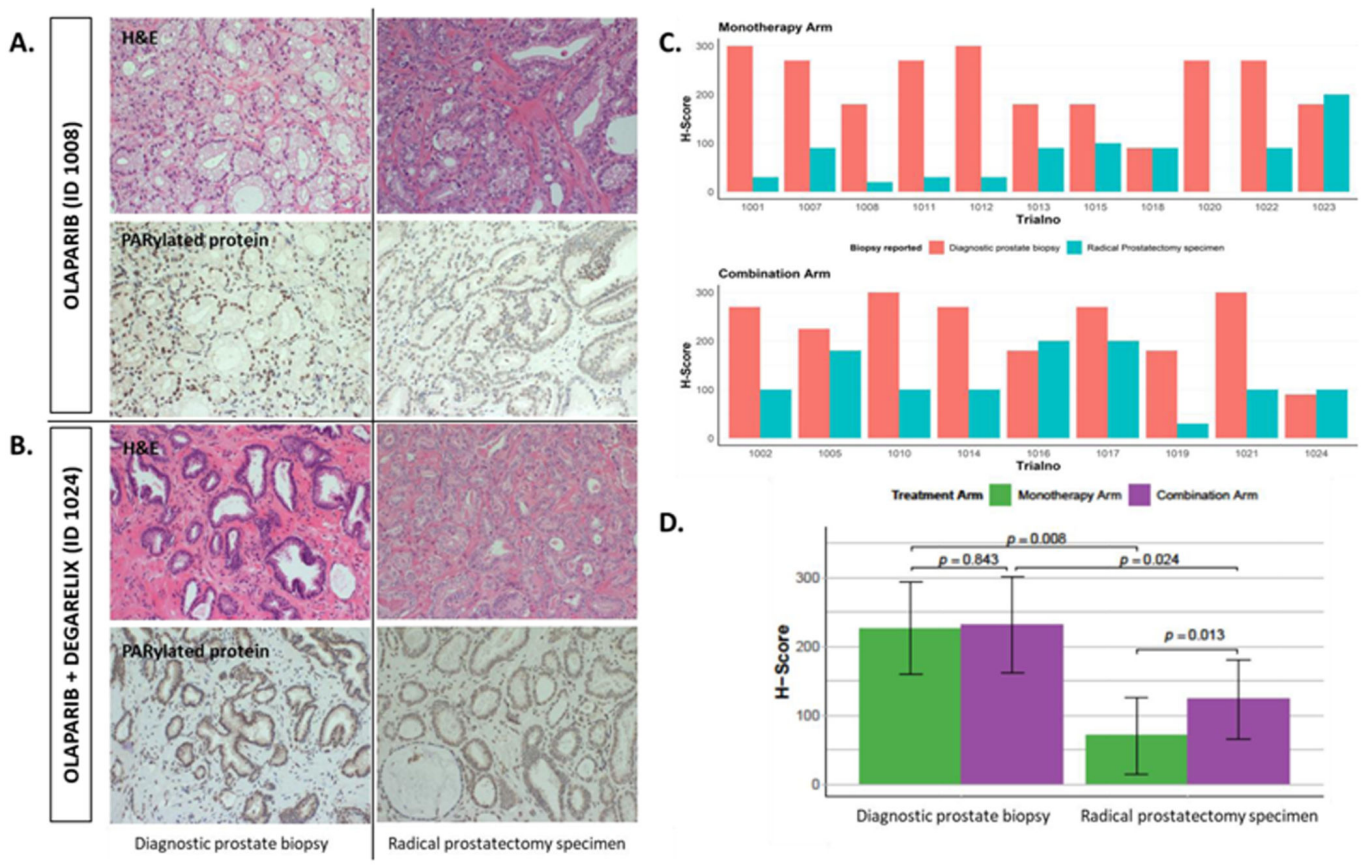
Representative IHC sections (x400 magnification) stained with H&E and for PAR protein expression at baseline (diagnostic prostate biopsy) compared to after treatment (radical prostatectomy specimen). A. olaparib and B. olaparib + degarelix. C. PAR protein expression, H-score, for individual participant samples measured before (diagnostic) and after (prostatectomy) olaparib monotherapy or olaparib + degarelix combination treatment. D. Mean H score for olaparib monotherapy and olaparib+degarelix combination cohorts at baseline and after treatment.

PARylated (PAR) protein expression, by IHC, was detectable at baseline in all participants (Figure 2) and there was no significant difference (p=0.84) in baseline PAR H-score between the two cohorts. Olaparib monotherapy significantly reduced PARylated protein expression (Figure 2D), mean reduction in H-score of 156 (range -270 to 20, *p*=0.008). In comparison PARylated protein expression was reduced after olaparib plus degarelix therapy but, the change in H-score was smaller with a mean reduction of 108 (range -200 to 20, *p*=0.024). PARylated protein expression measured in prostatectomy samples was lower in the olaparib [mean H-score of 70 (range 0 to 200)] cohort compared to the olaparib + degarelix [mean H-score of 123 (range 30 to 200)] cohort (p=0.013). Figure 2D. There were three participants with a >90% reduction in H-score (proportion 27.3%, 95% CI 6.0% to 60.9%), all from the olaparib cohort. In contrast a > 90% reduction was not measured in any samples from participants on the combination cohort (proportion 0% (95% CI: 0%, 33.6%).

### Safety and feasibility

Twenty-three participants were evaluable for safety analysis (one participant opted for radiotherapy and did not start IMP). A total of 21 (91.3%) participants experienced an AE during the study, 10 in the olaparib cohort and 11 in the olaparib + degarelix cohort. These AEs were mostly Grade 1 and Grade 2 and were in keeping with the known safety profile for both drugs.

In the olaparib cohort 6 AEs (17.6%) were related (definitely, probably or possibly) to olaparib, and 16 (47.1%) were related to surgery. In the olaparib + degarelix cohort, there were 22 (48.9%) AE related to degarelix, 7 (15.6%) related to surgery and 17 related to olaparib (37.8%). Supplementary Table 3 reports the AE by Grade for participants in both cohorts, no drug related AE ≥ Grade 3 were reported.

At the 6-weeks post-surgery (end of study) visit there were no unresolved AE in the olaparib cohort, three participants reported AE in the olaparib + degarelix cohort (weight loss, flushing, and hot flashes).

The majority of participants had surgery on the planned date (17, 85%). There were short delays in planned surgery date for three participants. Reasons were unrelated to IMP and included hospital scheduling (1 day), participant holiday (6 days) and recovery from accidental fall (7 days).

### PSA measurements

**Figure 3:**
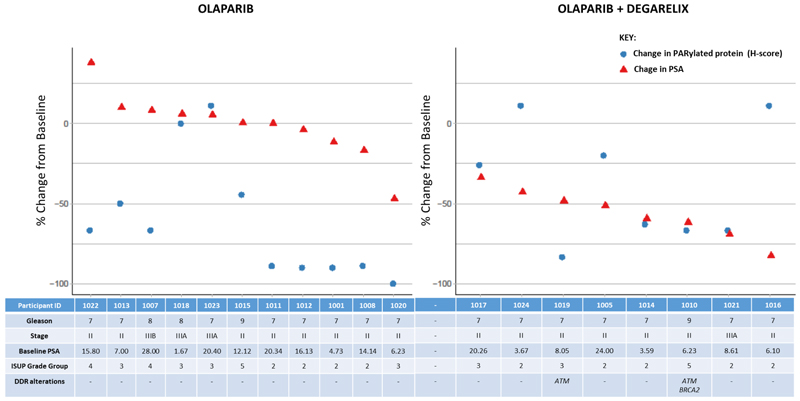
Left: Graph with individual participants, treated with olaparib, ordered by degree of change/ fall in PSA plotted with % change PAR expression (H-score), and significant DDR genetic alterations listed per participant. Right: Graph with individual participants, treated with olaparib + degarelix; ordered by degree of change/ fall in PSA plotted with % change PAR expression. Bottom summary table of clinical features (based on diagnostic biopsy/ baseline staging) per patient including significant genetic changes listed per participant – pathogenic variants in BRCA1, BRCA2 or ATM marked. NB. Participant 1002 did not have a PSA result from the day of radical prostatectomy.

PSA changes (% from baseline) were varied for individual participants given olaparib monotherapy, the maximum fall over two weeks was 47.1% (participant 1020). PSA falls were more consistent after olaparib + degarelix however, PSA changes of this magnitude and speed would be consistent with the effects of degarelix^36^ and the relative contribution of each agent is uncertain given the lack of single agent degarelix cohort(see Figure 3 and Supplementary Figure 1). A summary of all PSA and PARylation results is presented in Supplementary Table 4.

Pathological review of radical prostatectomy samples indicated that some malignant glands appeared shrunken and less indistinct, raising the possibility of morphological changes consistent with a treatment effect in participants 1002, 1008 and possibly 1016.

### Genome sequencing analysis

Retrospective tumour genetic analysis detected pathogenic HRR variants in only two participants, both randomised to combination olaparib and degarelix treatment. Participant 1019 carried a pathogenic somatic variant in *ATM* (R1875X) at a variant allele frequency (VAF) of 3.6%. Participant 1010 carried a germline variant in BRCA2 (S1982fs), alongside two somatic ATM variants (R250X, VAF of 4.3%, and R1730X, VAF of 3.4%), all three of which were classified as pathogenic in ClinVar. The reduction in PARylated protein expression of these two participants were among the largest changes measured in this arm (Figure 3). The complete list of pathogenic gene alterations identified is summarised in Supplementary Table 5. Somatic pathogenic variants were identified at frequencies between 2.3%-33.5%, indicative of the range of tumour purity achieved and the detection of subclonal variants.

### RNA sequencing analysis

**Figure 4:**
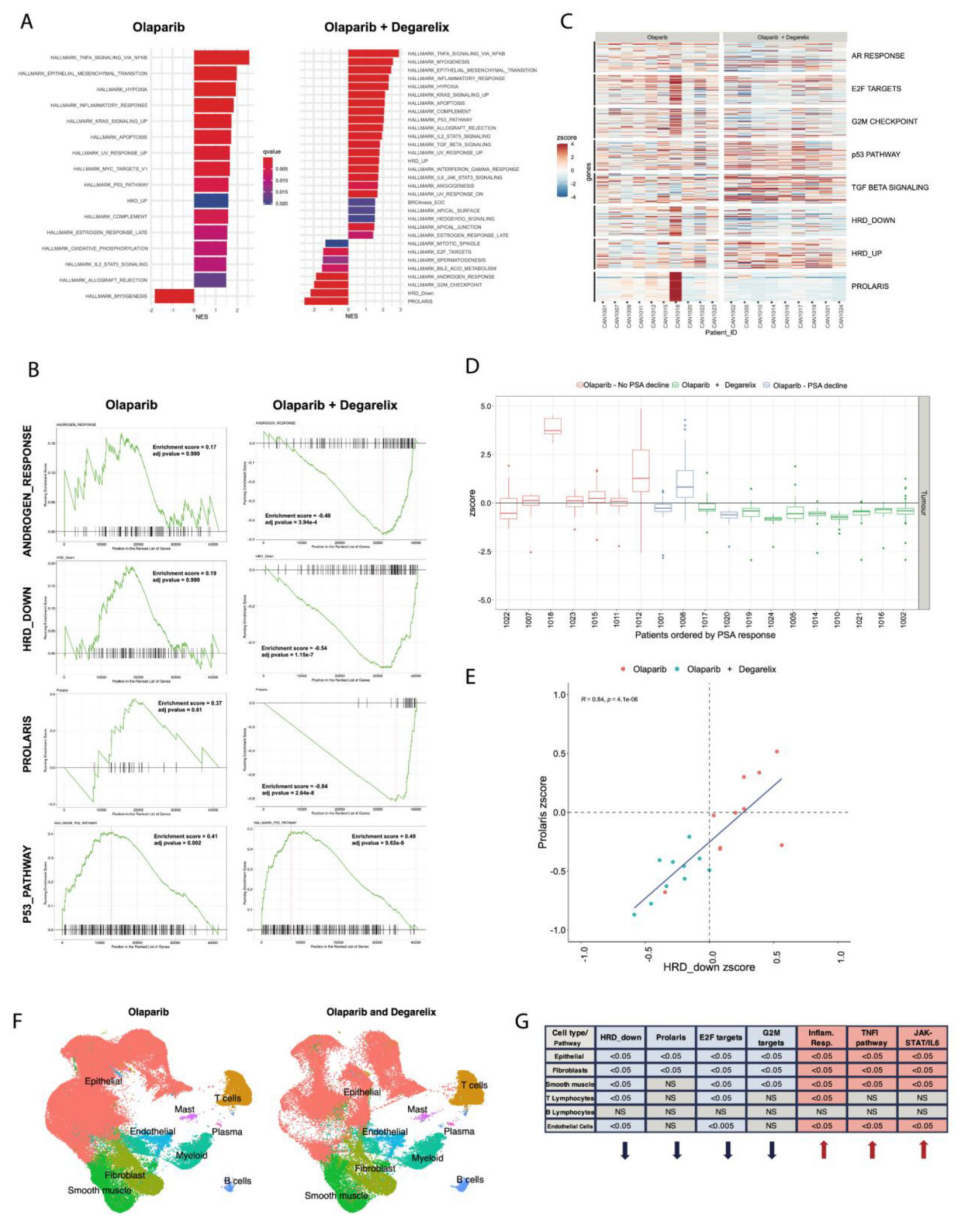
Transcriptomic analysis of CANCAP03 tumours. (A) Gene set enrichment analysis for participants treated with olaparib and olaparib + degarelix cohorts. Genes were ranked by Log2Fold change, with respect to pre-treatment samples. Enrichment analysis was performed using the MSigDB cancer hallmarks and expression signatures relating to homologous recombination deficiency (see supplementary for details). (B) Gene set enrichment plots revealing the direction of modulation for signatures of interest. (C) Heatmap showing the magnitude of expression changes across the participant cohorts for pathways of interest. Patient 1013 excluded from RNAseq analysis due to insufficient tumour content available. (D) Expression changes of Prolaris signatures following treatment, expressed as a Z-score with respect to pre-treatment samples, and ordered by PSA change. Olaparib either PSA decline (reduction > 10% from pre-treatment) or not and olaparib + degarelix (all participants had PSA decline). (E) Correlation between Prolaris and HRD down expression changes (with removal of outlier patient 1018). (F) Cell type annotation using single-nuclear RNA sequencing of n=6 patients from both monotherapy and combination treatment group. (G) Gene set enrichment analysis showing differences between olaparib + degarelix and olaparib treatment groups in the labelled pathways; arrows denote the trend of expression as upregulated (arrow up) or downregulation (arrow down), while numbers represent q values (NS – not significant).

We performed exploratory analysis using RNA sequencing on pre- and post-treatment tumour specimens. We performed Gene Set Enrichment Analysis (GSEA) using cancer hallmark gene sets, HR repair signatures and the prostate-specific cell cycle Prolaris signature (Figure 4).

The GSEA results for each treatment cohort demonstrated olaparib-related treatment response in cancer hallmark gene signatures (q<0.05) (Figure 4A). This included TNFa signalling via NFKB, EMT, hypoxia, inflammatory pathway upregulation, and myogenesis downregulation. We further observed an upregulation of the p53 pathway hallmark, which was more pronounced in the context of drug combination (Figure 4B). As anticipated, the combination treatment led to a downregulation of androgen response genes, attributable to the blockade of the androgen signalling axis by degarelix (Figure 4B). Combination treatment was also associated with increased activity of TNFa signalling via NFKB, myogenesis, EMT, inflammatory and hypoxia pathways. Additionally, we observed a suppression of canonical cell cycle progression hallmarks, including E2F targets and the G2M checkpoint, along with a significant downregulation of the Prolaris-based cell cycle progression signature (Figure 4C). Differential gene expression between olaparib + degarelix and olaparib is shown in Supplementary Figure 2A.

Evaluation of gene expression changes across the participant cohort revealed heterogeneous responses, particularly within the olaparib single-agent treatment cohort (Figure 4C). Patient ‘Prolaris’-related scores were mapped as a function of PSA change (Figure 4D). Amongst olaparib monotherapy treated patients, two of three patients with greatest PSA decline demonstrated large reductions in ‘Prolaris’ score. While patient 1018 (Stage T3a at study entry, no long term follow up data available) in the olaparib monotherapy cohort demonstrated the greatest Prolaris score, the change in PSA and PAR after treatment was unremarkable. Follow-up data was available for 8 of 20 patients; patient 1012 and 1015 (both pT3a with focal extracapsular extension) demonstrated a clinical relapse within 4 years (biochemical recurrence and metastatic disease respectively). Patients 1012 and 1015 also demonstrated greater ‘Prolaris’ scores amongst their, monotherapy, cohort.

We observed a significant positive correlation (R=0.84, p<0.05) between HR deficiency and prostate cancer proliferation index observed across the study cohorts (Figure 4E). There was minimal overlap in the genes constituting these two signatures (Supplementary Figure 2B). The pathways enriched in response to treatment remained the same, when excluding HR deficient patients 1010 and 1019 (Supplementary Figure 2C). This relationship was also observed following analysis of samples from a separate window of opportunity study cohort (DARANA trial^37^) in which patients were treated with enzalutamide alone (Supplementary Figure 2D-E).

We sought to validate the transcriptional changes within the tumour epithelial compartment itself, and applied single-nuclear RNA sequencing on a subset, where samples were available (n=6), of 3 patients from the monotherapy and 3 from the combination treatment groups. Sample integration and clustering was performed, followed by major cell type annotations (Supplementary Figure 3A-C), revealing a broadly consistent distribution of cell types across the 6 patients and between the 2 groups (Supplementary Figure 3D). When comparing olaparib + degarelix to the olaparib cohort, we were able to validate the Gene set enrichment analysis pathways highlighted from bulk RNA sequencing data. Furthermore, an increase in inflammatory response signalling, IL-6 and T-cell activity, was observed with combined androgen and PARP inhibition (Figure 4G).

## Discussion

The CANCAP03 trial successfully recruited men for a two-week course of olaparib or olaparib with degarelix before radical prostatectomy. The study achieved its primary endpoint, confirming a significant reduction in PARylated protein expression in prostate cancer cells – a pharmacodynamic biomarker of PARP inhibition after treatment. The short course of olaparib or olaparib plus degarelix were well tolerated by all participants and had no impact on planned prostatectomy or subsequent recovery.

The study’s short treatment period makes observing a treatment response less likely, especially in the (biomarker unselected) olaparib cohort. Pathology review of radical prostatectomy samples indicated a potential morphological treatment effect in participants 1002, 1008 and possibly 1016; interestingly, the PSA change (fall) in participants 1008 (olaparib) and 1016 (olaparib + degarelix) were among the largest in their respective cohorts. PSA data for participant 1002 was not available. We acknowledge the need for cautious interpretation of changes in PSA levels, nevertheless, there were some notable observations. As expected, PSA levels decreased significantly in participants treated with olaparib + degarelix due to effective androgen deprivation. In contrast, the single-agent olaparib arm had a more limited effect on PSA levels, but there were reductions noted in some patients. Intriguingly, participants with the most substantial reduction in PARylated protein expression after olaparib treatment also had stable or falling PSA levels, consistent with previous studies that link the degree of PARP inhibtion with anti-cancer effect ^38^. We did not observe a similar trend in the analysis of samples from participants treated with olaparib + degarelix, likely due to the dominant effect of androgen blockade on overall PSA levels although two participants with *BRCA2* and/or *ATM* variants exhibited some of the largest reductions in PARylated protein expression within the cohort (patients 1010 and 1019).

Our transcriptomic analysis revealed a significant reduction in proliferation and activation of cell cycle checkpoints in response to combination treatment. There was a general trend observed between PSA response and the ‘Prolaris’ signature, with a few exceptions within the olaparib monotherapy cohort. In follow up data from one trial site (where data was available), two out of eight participants had experienced progression within four years of radical prostatectomy. Participant 1012 had biochemical recurrence within four years, and participant 1015 developed metastatic disease, both participants had pT3a disease with focal extra prostatic extension. These adverse disease features and outcomes may be related to higher cellular turnover. While previous research has suggested an interplay between PARP1 and the regulation of AR-mediated gene expression^12^, we did not observe significant consequences on the androgen signalling axis resulting from PARP inhibition with olaparib monotherapy.

Interestingly, the reduction in PARylated protein expression appeared more pronounced in tumour samples from participants treated with olaparib alone compared to olaparib + degarelix. These results might suggest co-inhibition of PARP and AR pathways may be less effective at inhibiting PARylation compared to single agent PARP inhibition with olaparib. The greater PARylated protein expression might be due to more abundant substrate availability i.e. more DNA damage sensing and binding by PARP catalysing its post translational polymerisation of PARs onto target proteins; thus while the baseline PARP inhibition is the same, activation is greater following the DNA damage associated with androgen deprivation..

Transcriptomic profiling also revealed a signature previously associated with HR deficiency, which appeared to correspond directly to reductions in cellular proliferation (i.e. ‘Prolaris’). This trend was observed in the combination treatment cohort and in the analysis of data from a separate androgen pathway inhibitor monotherapy study. Previous experimental work in cell lines has demonstrated a reduction in DNA repair gene expression resulting from androgen inhibition^39–41^ potentially leading to a clinical demonstration of induced BRCAness. However, it is unclear whether the HRD-associated signature changes are a direct effect of treatment or an indirect consequence of proliferation inhibition and cell cycle alterations^42^. This may suggest alternative mechanisms of action rather than an anti-androgen induced BRCAness state. Further studies will be required in order to validate the significance of these tissue responses.

Our study revealed several unique hallmarks in response to combination treatment that were not as strongly observed with olaparib monotherapy, or in a separate neoadjuvant study involving anti-androgen monotherapy. Upregulation of TGF-β, allograft rejection and apical junction pathways may suggest additional transcriptional, immunological or cell differentiation processes involved in supporting the AR-mediated DNA damage response. Further mechanistic studies will be needed to explain these unique upstream cellular processes and cofactors that contribute to the AR-supported DNA damage response in the context of PARP inhibition. We also observed a higher inflammatory response hallmark observed following the addition of degarelix to olaparib, including TNFα and JAK-STAT signalling, including within tumour epithelial fractions. It remains to be seen whether greater treatment-induced inflammatory response signalling could sensitise tumours to immune-targeting strategies.

Our study had limitations, primarily the design was pragmatic, using a short treatment course, in order to balance participants safety, avoiding any impact on planned curative surgery. We sought to minimise additional interventions for participants where possible, in order to maximise recruitment. These decisions limited some opportunities for additional research, for example relying on the pre-existing diagnostic (FFPE) prostate biopsy as baseline sample. A longer duration of olaparib treatment may be required for anti-cancer effect, which would also be simpler to detect robustly over a longer follow up period. Unfortunately, schedule changes in the date of radical prostatectomy led to three participants not being evaluable, although they were replaced per protocol. We acknowledge a degarelix monotherapy control arm was lacking, as CANCAP03 was an investigator-initiated trial we had planned to incorporate data from a previous study of degarelix, NCT01852864, which was not possible given the analytical methods differing between studies. However, we believe these are the still the first, detailed, prospective data to be reported from a cohort of men with localised prostate cancer treated with PARPi and castration.

Ongoing studies by other groups, such as those using niraparib (NCT04030559) might extend our initial observations by testing longer IMP durations with alternative trial endpoints or by collecting additional data, such as the NePtune study of olaparib in men with DDR mutations (NCT05498272). Peri-operative studies remain challenging, as we note a study with extended olaparib treatment was terminated early due to slow recruitment (NCT03570476).

In conclusion, our study established a well-tolerated window trial design and supported the use of short duration PARP inhibition with or without ADT to study the biology of these agents in men with localised prostate cancer. Evidence suggests that PARP inhibition is a promising treatment for men with DNA repair gene defects, while the role of combined treatment with ARSI in genetically unselected men remains a topic of debate.

To our knowledge, these data represent the first prospective report detailing transcriptomic changes following a short course of combined PARP and androgen inhibition. We anticipate that additional studies (e.g. ASCERTAIN, NCT05938270), informed by the lessons learned from this exploratory study, will provide additional insights, ultimately improving the outcomes for men with prostate cancer.

## Supplementary Material

1

2

3

4

Supplementary Information

## Figures and Tables

**Table 1:** Summary of participant demographics at registration, based on diagnostic needle biopsy (patients evaluable for primary endpoint, see Supplementary Table 2 for all patients demographics)

		Olaparib (n=11)	Olaparib + Degarelix (n=9)
**Age, years**			
	Median (range)	60 (47-71)	63 (49-69)
**Ethnicity**			
	White	11 (100%)	7 (77.8%)
	Asian/ Asian British	0	1 (11.1%)
	Other	0	1 (11.1%)
**Performance Status (ECOG)**			
	0	11 (100%)	8 (88.9%)
	1	0	1 (11.1%)
**Histology (ISUP Grade Group)**			
	2	4 (37%)	5 (56%)
	3	3 (27%)	3 (33%)
	4	3 (27%)	0
	5	1 (9%)	1 (11%)
**T-Stage**			
	2	8 (72.7%)	8 (88.9%)
	3a	2 (18.2%)	1 (11.1%)
	3b	1 (9.1%)	0
**D’Amico Risk**			
	Intermediate	5 (45.5%)	6 (66.7%)
	High	6 (54.5%)	3 (33.3%)

## Data Availability

The data generated in this study are available within the article and its supplementary data files. The sequence data generated in this study are publicly available in the European Genome-Phenome Archive (EGAS50000000880, EGAD50000001280) and within the article and its supplementary data files. The data from the Broad Institute’s Cancer Dependency Map project that were analyzed in this study were obtained from figshare at https://doi.org/10.6084/m9.figshare.12280541.v4.

## Translational Relevance

To our knowledge CANCAP03 is the first neoadjuvant, randomised study of PARP inhibitor ± androgen deprivation to be reported. Recruiting men, prior to radical prostatectomy, was safe, acceptable to participants and the study design allowed analysis of human prostate cancer tissue after drug treatment. We confirmed effective PARP inhibition and report detailed transcriptomic analysis at bulk and single cell resolution. These insights are critical in order to better understand the clinical synergy reported in Phase 3 trials of PARPi and androgen deprivation for patients with tumours that do not harbour defects in DNA repair pathways given that the proposed mechanisms are, to date, based on pre-clinical studies.
