# Supplementary material for: Cambridge Neoadjuvant Cancer of the Prostate (CANCAP03): A Window Study into the Effects of Olaparib ± Degarelix in Primary Prostate Cancer
Source: Clin Cancer Res. Author manuscript; Available in PMC 2025 Jun 23. (PMC7617790; doi:10.1158/1078-0432.CCR-24-1304)
Supplement: 2 [file EMS204853-supplement-2.pptx]

## Slide 1
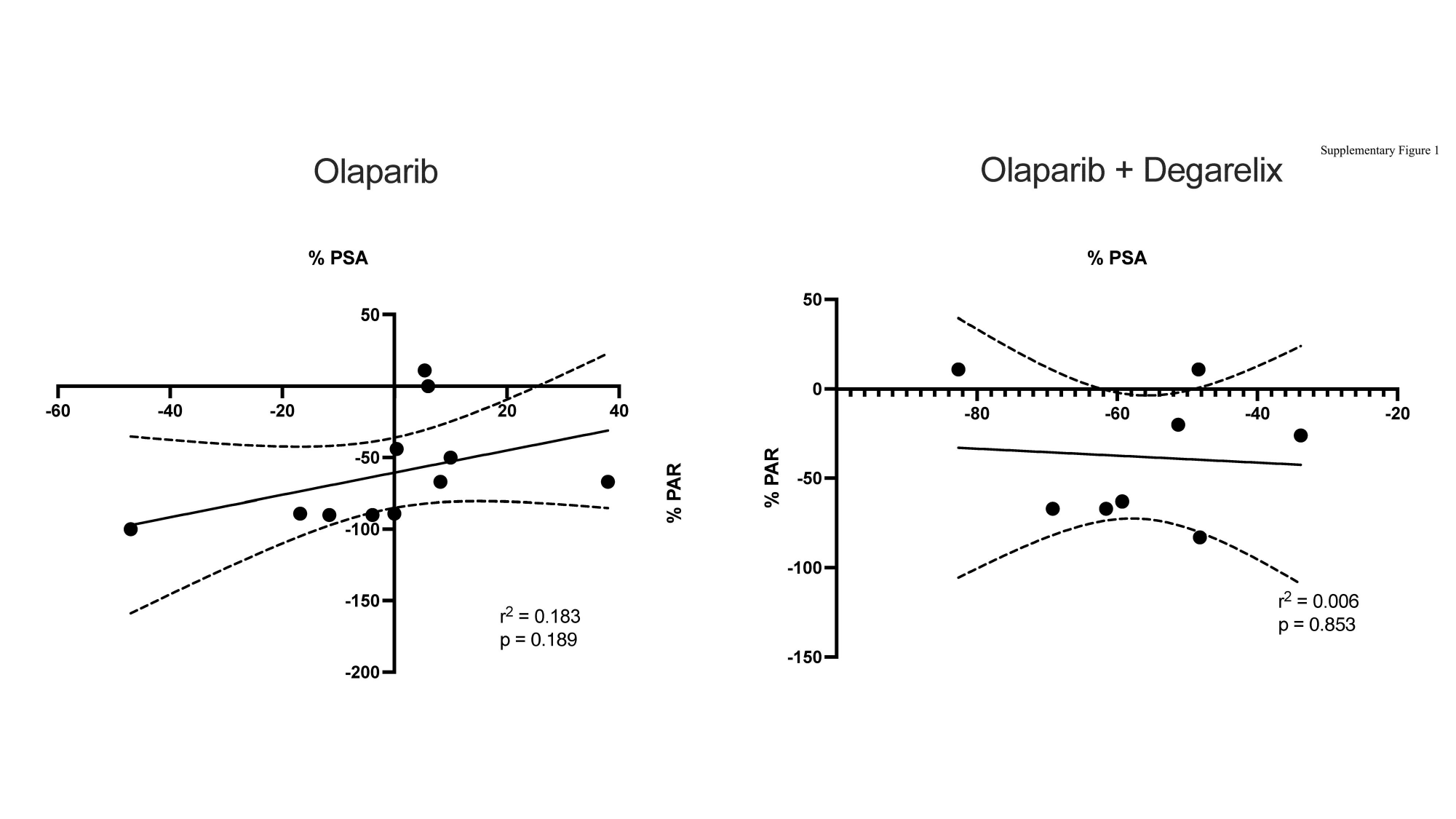

## Slide 2
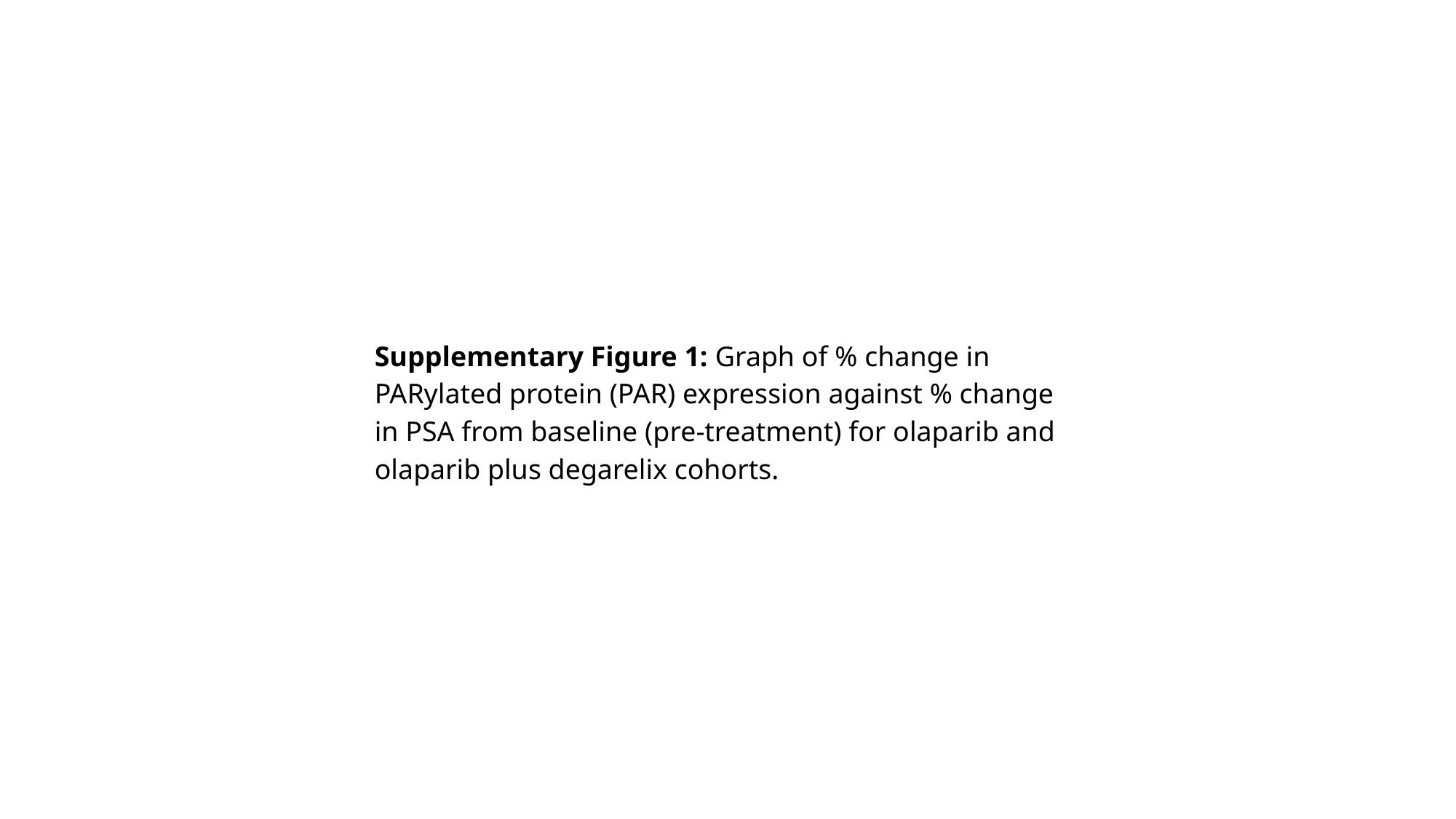

Supplementary Figure 1: Graph of % change in PARylated protein (PAR) expression against % change in PSA from baseline (pre-treatment) for olaparib and olaparib plus degarelix cohorts.
