# Supplementary material for: Cambridge Neoadjuvant Cancer of the Prostate (CANCAP03): A Window Study into the Effects of Olaparib ± Degarelix in Primary Prostate Cancer
Source: Clin Cancer Res. Author manuscript; Available in PMC 2025 Jun 23. (PMC7617790; doi:10.1158/1078-0432.CCR-24-1304)
Supplement: 3 [file EMS204853-supplement-3.pptx]

## Slide 1
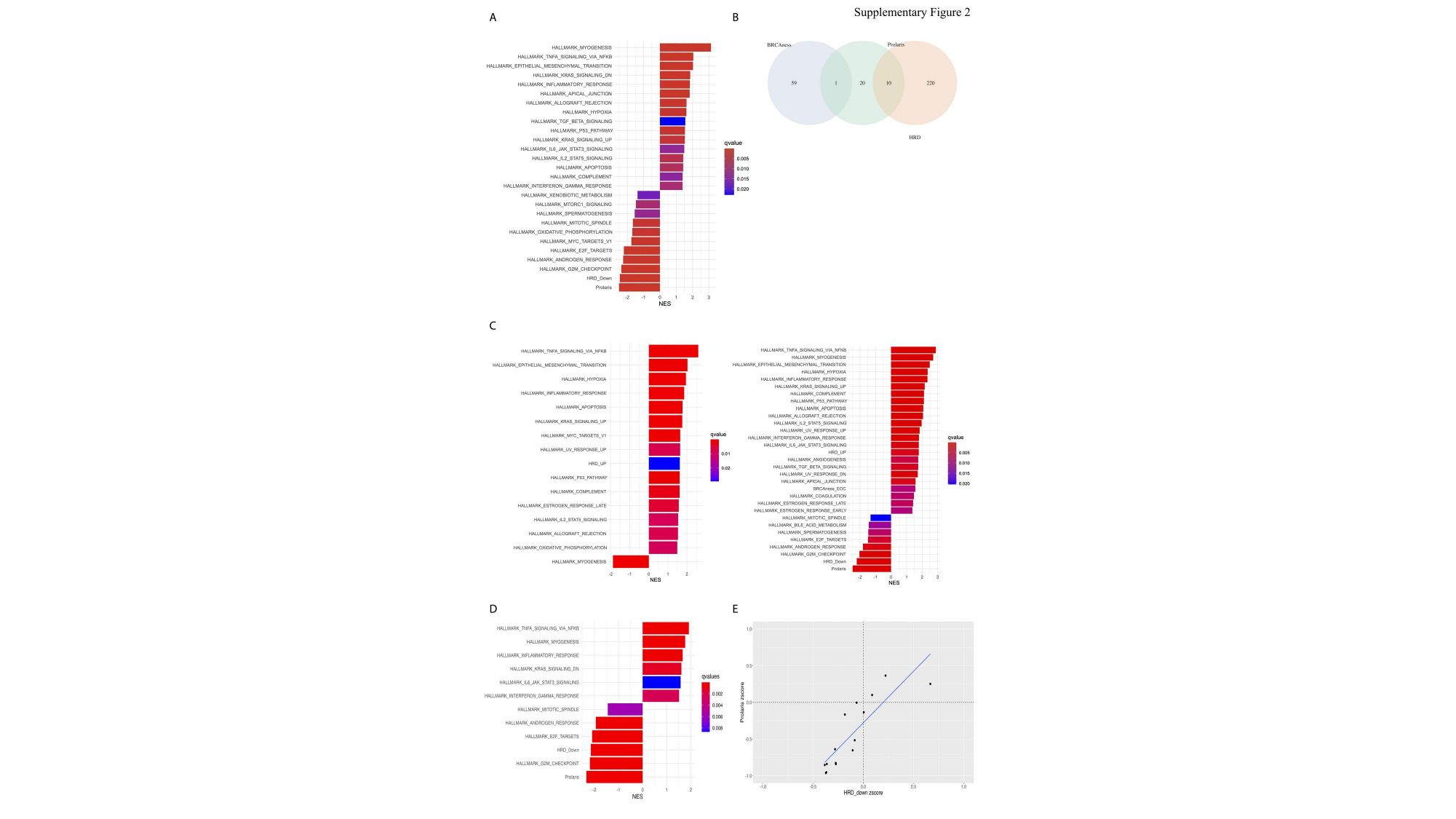

## Slide 2
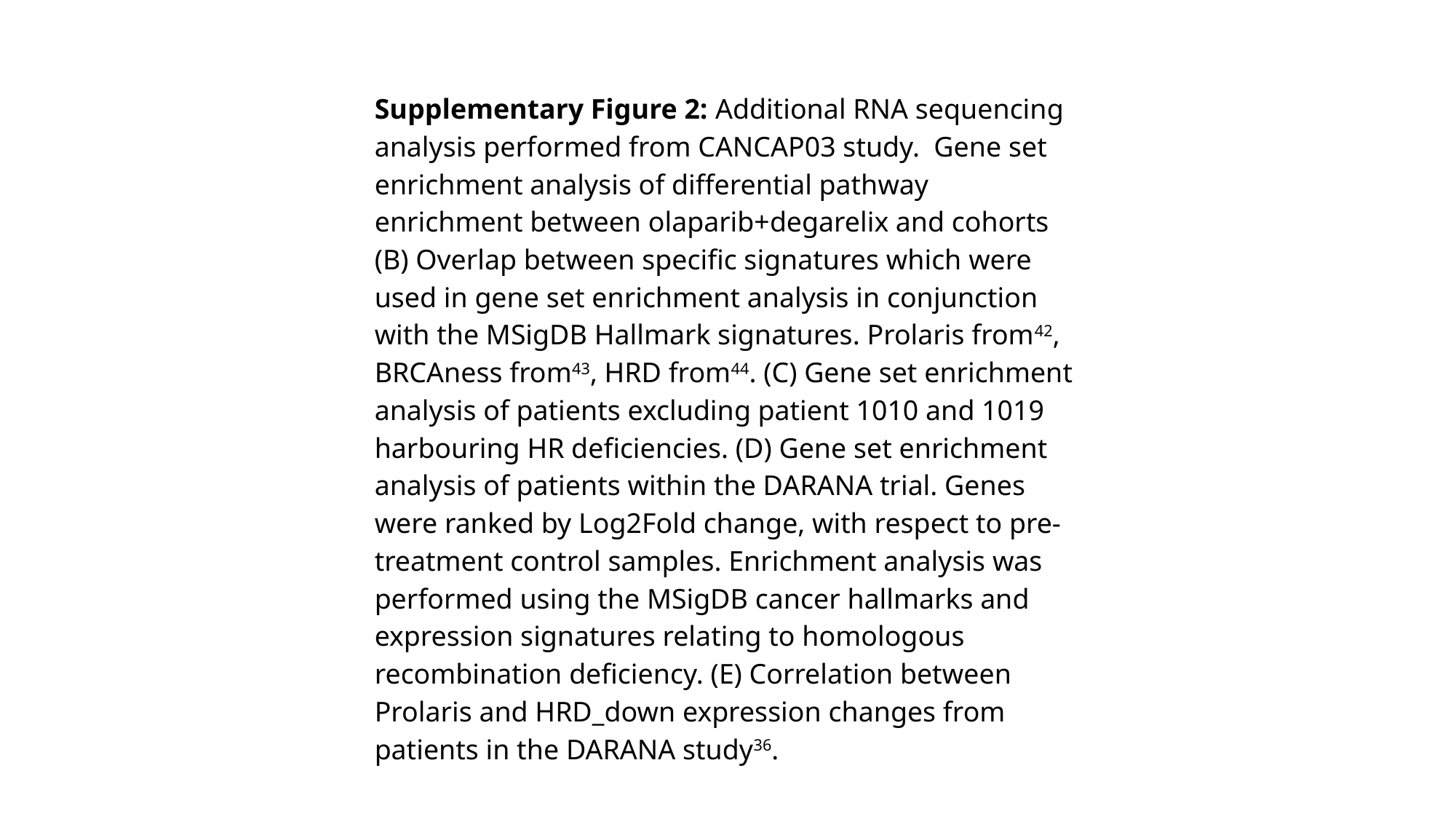

Supplementary Figure 2: Additional RNA sequencing analysis performed from CANCAP03 study. Gene set enrichment analysis of differential pathway enrichment between olaparib+degarelix and cohorts (B) Overlap between specific signatures which were used in gene set enrichment analysis in conjunction with the MSigDB Hallmark signatures. Prolaris from42, BRCAness from43, HRD from44. (C) Gene set enrichment analysis of patients excluding patient 1010 and 1019 harbouring HR deficiencies. (D) Gene set enrichment analysis of patients within the DARANA trial. Genes were ranked by Log2Fold change, with respect to pre-treatment control samples. Enrichment analysis was performed using the MSigDB cancer hallmarks and expression signatures relating to homologous recombination deficiency. (E) Correlation between Prolaris and HRD_down expression changes from patients in the DARANA study36.
