# Supplementary material for: Cambridge Neoadjuvant Cancer of the Prostate (CANCAP03): A Window Study into the Effects of Olaparib ± Degarelix in Primary Prostate Cancer
Source: Clin Cancer Res. Author manuscript; Available in PMC 2025 Jun 23. (PMC7617790; doi:10.1158/1078-0432.CCR-24-1304)
Supplement: 4 [file EMS204853-supplement-4.pptx]

## Slide 1
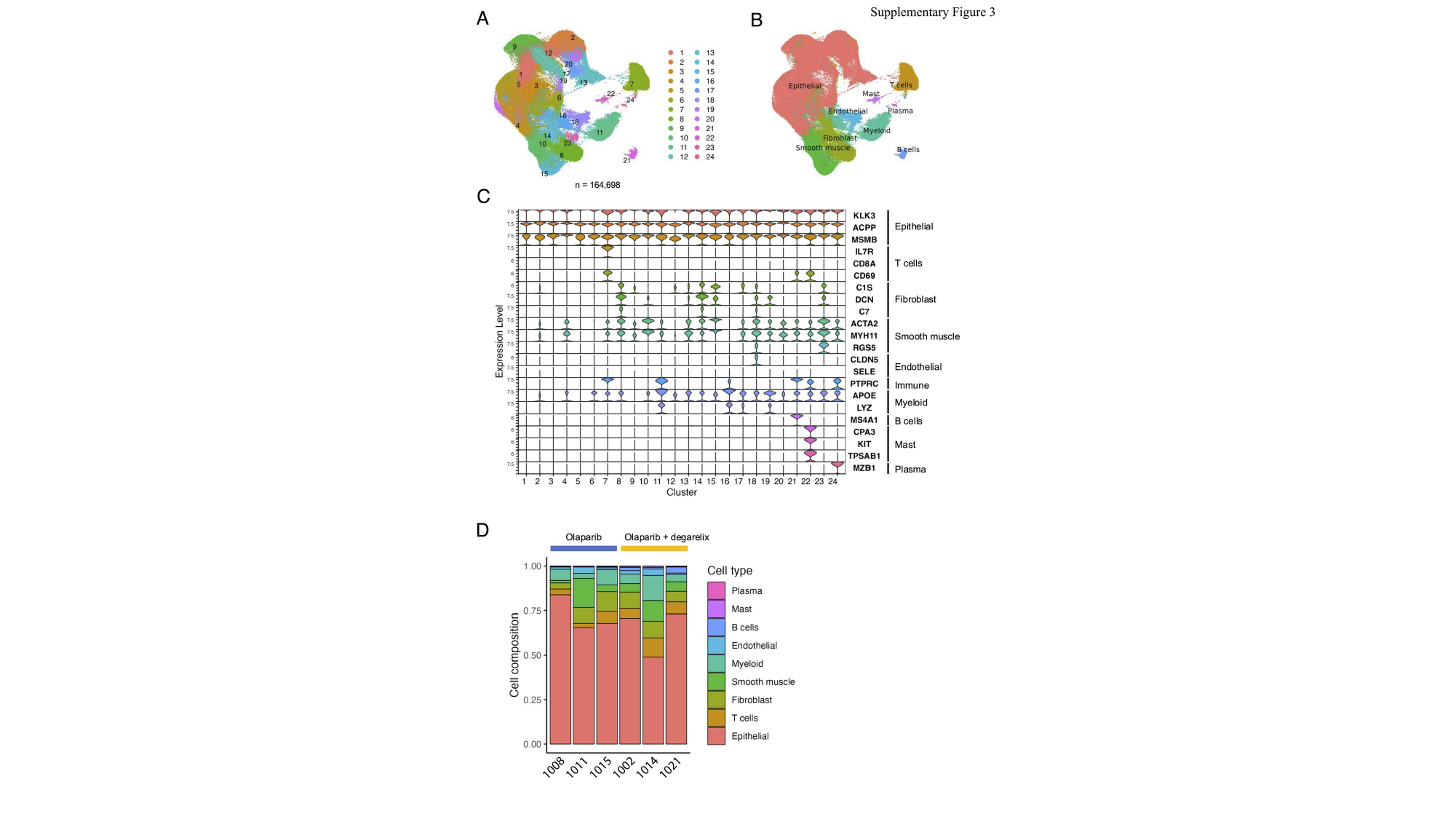

## Slide 2
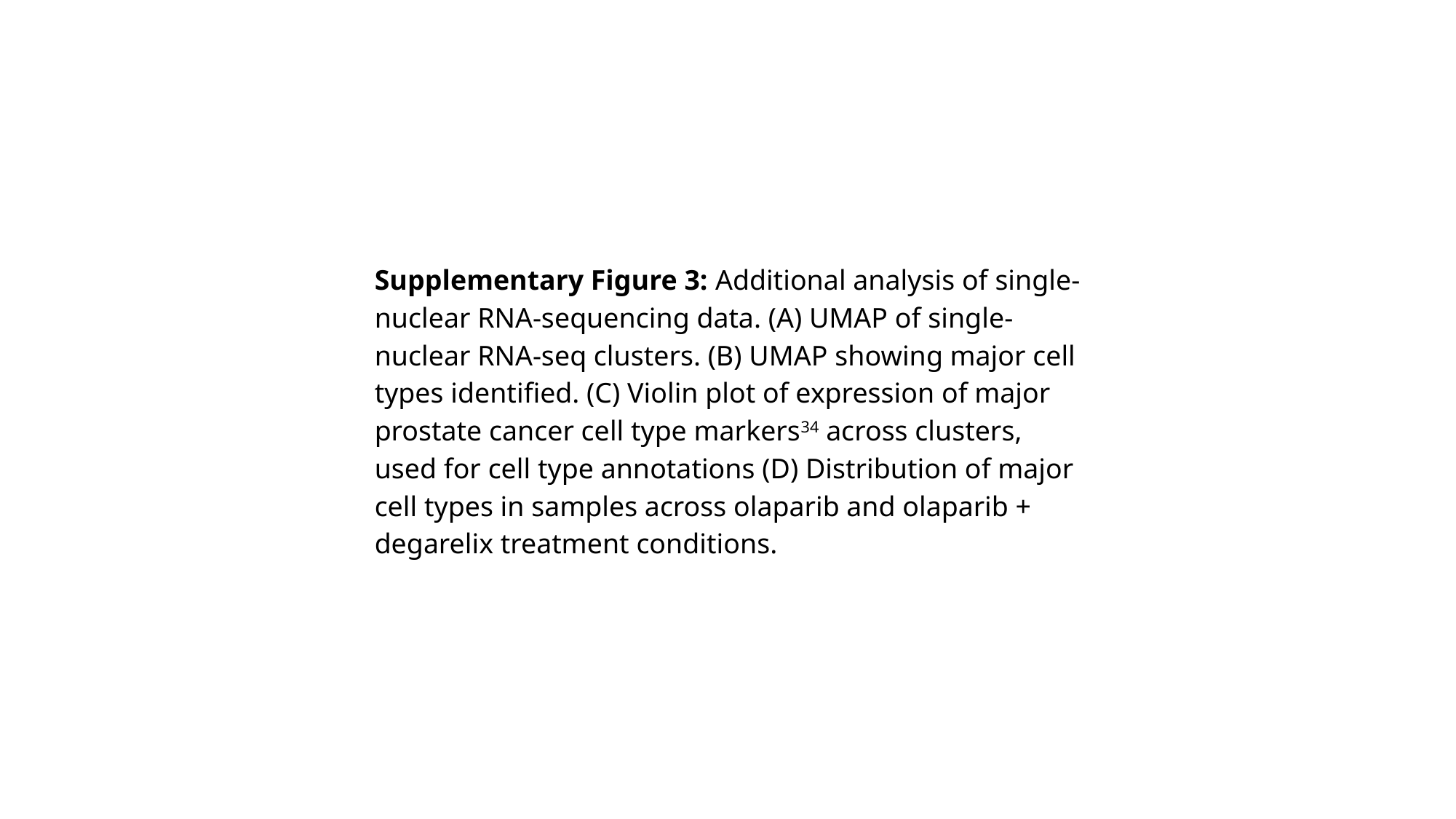

Supplementary Figure 3: Additional analysis of single-nuclear RNA-sequencing data. (A) UMAP of single-nuclear RNA-seq clusters. (B) UMAP showing major cell types identified. (C) Violin plot of expression of major prostate cancer cell type markers34 across clusters, used for cell type annotations (D) Distribution of major cell types in samples across olaparib and olaparib + degarelix treatment conditions.
