# Supplementary material for: Cambridge Neoadjuvant Cancer of the Prostate (CANCAP03): A Window Study into the Effects of Olaparib ± Degarelix in Primary Prostate Cancer
Source: Clin Cancer Res. Author manuscript; Available in PMC 2025 Jun 23. (PMC7617790; doi:10.1158/1078-0432.CCR-24-1304)
Supplement: Supplementary Information [file EMS204853-supplement-Supplementary_Information.docx]

**Supplementary Table 1: List of genes covered in targeted sequencing and mutation panel**

| **Gene symbol** | |  |  |  |  |  |  |  |  |  |  |  |  |  |
| --- | --- | --- | --- | --- | --- | --- | --- | --- | --- | --- | --- | --- | --- | --- |
| ABL1 | BAP1 | CD274 | CUL3 | ERCC3 | FH | HNF1A | KDM5A | MED12 | NFE2L2 | PHOX2B | PTPN11 | RIT1 | SOCS1 | TP53 |
| AKT1 | BARD1 | CD276 | DAXX | ERCC4 | FLCN | HRAS | KDM5C | MEF2B | NKX2-1 | PIK3C2G | PTPRD | RNF43 | SOX17 | TP63 |
| AKT2 | BBC3 | CD79B | DCUN1D1 | ERCC5 | FLT1 | ICOSLG | KDM6A | MEN1 | NKX3-1 | PIK3C3 | PTPRS | ROS1 | SOX2 | TRAF7 |
| AKT3 | BCL2 | CDC73 | DDR2 | ERG | FLT3 | IDH1 | KDR | MET | NOTCH1 | PIK3CA | PTPRT | RPS6KA4 | SOX9 | TSC1 |
| ALK | BCL2L1 | CDH1 | DICER1 | ESR1 | FLT4 | IDH2 | KEAP1 | MITF | NOTCH2 | PIK3CB | RAC1 | RPS6KB2 | SPEN | TSC2 |
| ALOX12B | BCL2L11 | CDK12 | DIS3 | ETV1 | FOXA1 | IFNGR1 | KIT | MLH1 | NOTCH3 | PIK3CD | RAD50 | RPTOR | SPOP | TSHR |
| APC | BCL6 | CDK4 | DNMT1 | ETV6 | FOXL2 | IGF1 | KLF4 | MLL | NOTCH4 | PIK3CG | RAD51 | RUNX1 | SRC | U2AF1 |
| AR | BCOR | CDK6 | DNMT3A | EZH2 | FOXP1 | IGF1R | KRAS | MLL2 | NPM1 | PIK3R1 | RAD51B | RYBP | STAG2 | VHL |
| ARAF | BLM | CDK8 | DNMT3B | FAM123B | FRS2 | IGF2 | LATS1 | MLL3 | NRAS | PIK3R2 | RAD51C | SDHA | STK11 | VTCN1 |
| ARID1A | BMPR1A | CDKN1A | DOT1L | FAM175A | FUBP1 | IKBKE | LATS2 | MPL | NSD1 | PIK3R3 | RAD51D | SDHAF2 | STK40 | WT1 |
| ARID1B | BRAF | CDKN1B | E2F3 | FAM46C | GATA1 | IKZF1 | LMO1 | MRE11A | NTRK1 | PIM1 | RAD52 | SDHB | SUFU | XIAP |
| ARID2 | BRCA1 | CDKN2A | EED | FANCA | GATA2 | IL10 | MAD2L2 | MSH2 | NTRK2 | PLK2 | RAD54L | SDHC | SUZ12 | XPO1 |
| ARID5B | BRCA2 | CDKN2B | EGFL7 | FANCC | GATA3 | IL7R | MALT1 | MSH6 | NTRK3 | PMAIP1 | RAF1 | SDHD | SYK | YAP1 |
| ASXL1 | BRD4 | CDKN2C | EGFR | FANCL | GNA11 | INPP4A | MAP2K1 | MTOR | PAK1 | PMS1 | RARA | SETD2 | TBX3 | YES1 |
| ASXL2 | BRIP1 | CHEK1 | EIF1AX | FANCM | GNAQ | INPP4B | MAP2K2 | MUTYH | PAK7 | PMS2 | RASA1 | SF3B1 | TERT |  |
| ATM | BTK | CHEK2 | EP300 | FAT1 | GNAS | INSR | MAP2K4 | MYC | PALB2 | PNRC1 | RB1 | SH2D1A | TET1 |  |
| ATR | CARD11 | CIC | EPCAM | FBXW7 | GREM1 | IRAK4 | MAP3K1 | MYCL1 | PARK2 | POLD1 | RBM10 | SHQ1 | TET2 |  |
| ATRX | CASP8 | CREBBP | EPHA3 | FGF19 | GRIN2A | IRF4 | MAP3K13 | MYCN | PARP1 | POLE | RECQL4 | SMAD2 | TGFBR1 |  |
| AURKA | CBFB | CRKL | EPHA5 | FGF3 | GSK3B | IRS1 | MAPK1 | MYD88 | PAX5 | PPP2R1A | REL | SMAD3 | TGFBR2 |  |
| AURKB | CBL | CRLF2 | EPHB1 | FGF4 | H3F3C | IRS2 | MAX | MYOD1 | PBRM1 | PPP2R2A | RET | SMAD4 | TMEM127 | |
| AXIN1 | CCND1 | CSF1R | ERBB2 | FGFR1 | HGF | JAK1 | MCL1 | NBN | PDCD1 | PRDM1 | RFWD2 | SMARCA4 | TMPRSS2 |  |
| AXIN2 | CCND2 | CTCF | ERBB3 | FGFR2 | HIST1H1C | JAK2 | MDC1 | NCOR1 | PDGFRA | PRKAR1A | RHEB | SMARCB1 | TNFAIP3 |  |
| AXL | CCND3 | CTLA4 | ERBB4 | FGFR3 | HIST1H2BD | JAK3 | MDM2 | NF1 | PDGFRB | PTCH1 | RHOA | SMARCD1 | TNFRSF14 | |
| B2M | CCNE1 | CTNNB1 | ERCC2 | FGFR4 | HIST1H3B | JUN | MDM4 | NF2 | PDPK1 | PTEN | RICTOR | SMO | TOP1 |  |

**Supplementary Table 2.** Baseline characteristics of all randomised participants

|  | | Olaparib (n=12) | Olaparib +Degarelix (n=12) |
| --- | --- | --- | --- |
| Age, years | |  |  |
|  | Median (range) | 60 (47-71) | 65 (49-72) |
| Ethnicity | |  |  |
|  | White | 12 (100%) | 9 (75.0%) |
|  | Asian/ Asian British | 0 | 2 (16.7%) |
|  | Other | 0 | 1 (8.3%) |
| Performance Status (ECOG) | |  |  |
|  | 0 | 12 (100%) | 11 (91.7%) |
|  | 1 | 0 | 1 (8.3%) |
| Histology (Gleason sum score) | |  |  |
|  | 7 | 9 (75.0%) | 10 (83.3%) |
|  | 8 | 2 (16.7%) | 0 |
|  | 9 | 1 (8.3%) | 2 (16.7%) |
| T-Stage | |  |  |
|  | 2 | 9 (75.0%) | 11 (91.7%) |
|  | 3a | 2 (16.7%) | 1 (8.3%) |
|  | 3b | 1 (8.3%) | 0 |
| D’Amico Risk | |  |  |
|  | Intermediate | 6 (50.0%) | 9 (75.0%) |
|  | High | 6 (50.0%) | 3 (25.0%) |

**Supplementary Table 3**: Summary of participant experienced AEs by grade

|  | **Olaparib** | | | | | | **Olaparib + Degarelix** | | | |
| --- | --- | --- | --- | --- | --- | --- | --- | --- | --- | --- |
|  | **Grade 1** | | **Grade 2** | | **Grade 3** | | **Grade 1** | | **Grade 2** | |
|  | **n** | **%** | **n** | **%** | **n** | **%** | **n** | **%** | **n** | **%** |
| **No. of participants experiencing AEs** | 8 | 66.7 | 5 | 41.7 | 2 | 16.7 | 10 | 90.9 | 5 | 45.5 |
| Fatigue | 2 | 16.7 |  |  |  |  | 6 | 54.5 |  |  |
| Constipation | 3 | 25 |  |  |  |  | 3 | 27.3 |  |  |
| Injection site reaction |  |  |  |  |  |  | 4 | 36.4 | 1 | 9.1 |
| Nausea | 2 | 16.7 |  |  |  |  | 2 | 18.2 | 1 | 9.1 |
| Hypertension |  |  | 3 | 25 | 1 | 8.3 |  |  | 1 | 9.1 |
| Urinary incontinence | 1 | 8.3 | 3 | 25 |  |  |  |  | 1 | 9.1 |
| Hot flashes |  |  |  |  |  |  | 4 | 36.4 |  |  |
| Vomiting | 1 | 8.3 |  |  |  |  | 2 | 18.2 | 1 | 9.1 |
| Erectile dysfunction |  |  | 2 | 16.7 |  |  |  |  | 1 | 9.1 |
| Anorexia |  |  |  |  |  |  | 1 | 9.1 | 1 | 9.1 |
| Diarrhea | 1 | 8.3 |  |  |  |  | 1 | 9.1 |  |  |
| Headache | 1 | 8.3 |  |  |  |  | 1 | 9.1 |  |  |
| Pain | 1 | 8.3 |  |  |  |  | 1 | 9.1 |  |  |
| Abdominal pain | 2 | 16.7 |  |  |  |  |  |  |  |  |
| Back pain |  |  |  |  |  |  | 1 | 9.1 |  |  |
| Chills |  |  |  |  |  |  | 1 | 9.1 |  |  |
| Creatinine increased |  |  |  |  |  |  | 1 | 9.1 |  |  |
| Dysgeusia |  |  |  |  |  |  | 1 | 9.1 |  |  |
| Gastroesophageal reflux disease |  |  |  |  |  |  | 1 | 9.1 |  |  |
| Lipohypertrophy |  |  |  |  |  |  | 1 | 9.1 |  |  |
| Pruritus |  |  |  |  |  |  | 1 | 9.1 |  |  |
| Weight loss |  |  |  |  |  |  | 1 | 9.1 |  |  |
| Flank pain |  |  |  |  |  |  |  |  | 1 | 9.1 |
| QTc Prologation |  |  |  |  |  |  |  |  | 1 | 9.1 |
| Urinary tract infection |  |  |  |  |  |  |  |  | 1 | 9.1 |
| Blood bilirubin increased | 1 | 8.3 |  |  |  |  |  |  |  |  |
| Depression | 1 | 8.3 |  |  |  |  |  |  |  |  |
| Penile pain | 1 | 8.3 |  |  |  |  |  |  |  |  |
| Upper respiratory infection | 1 | 8.3 |  |  |  |  |  |  |  |  |
| Wound dehiscence | 1 | 8.3 |  |  |  |  |  |  |  |  |
| Perineal pain |  |  | 1 | 8.3 |  |  |  |  |  |  |
| Skin and subcutaneous tissue  disorders - Other, groin rash |  |  | 1 | 8.3 |  |  |  |  |  |  |
| Wound infection |  |  | 1 | 8.3 |  |  |  |  |  |  |
| Urosepsis |  |  |  |  | 1 | 8.3 |  |  |  |  |

**
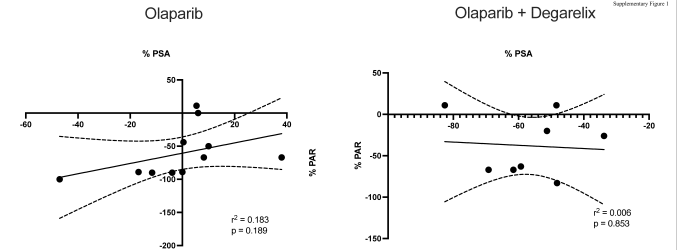
**

**Supplementary Figure 1:** Graph of % change in PARylated protein (PAR) expression against % change in PSA from baseline (pre-treatment) for olaparib and olaparib plus degarelix cohorts.

|  | **Drug(s)** | **Evaluable?** | **Baseline** | | | **Radical Prostatectomy** | | | | | **Follow up (6-weeks)** | |
| --- | --- | --- | --- | --- | --- | --- | --- | --- | --- | --- | --- | --- |
| **ID** |  |  | **PSA**  **ng/mL** | **Testosterone**  **nmol/L** | **PAR**  **H-Score** | **PSA**  **ng/mL** | **PSA**  **% baseline** | **PSA**  **% change** | **Testosterone**  **nmol/L** | **PAR**  **H-Score** | **PSA**  **ng/mL** | **Testosterone** |
| **1001** | O | Y | 4.73 | 12.1 | 300 | 4.18 | 88.4 | -11.6 | 8.7 | 30 | 0.1 | 16.8 |
| **1003** | O | N | 17.43 | 15.6 | - | - | - | - | - | - | 0.02 | 9.4 |
| **1006** | O | N | 7.15 | 14.2 | - | - | - | - | - | - | - | - |
| **1007** | O | Y | 28.0 | 21.4 | 270 | 30.3 | 108.2 | 8.2 | 16.1 | 90 | 0.1 | 20.3 |
| **1008** | O | Y | 14.14 | 19.4 | 180 | 11.77 | 83.2 | -16.8 | 16.7 | 20 | 0.03 | 20.2 |
| **1011** | O | Y | 20.34 | 25.4 | 270 | 20.35 | 100.0 | 0.0 | 16.1 | 30 | 0.02 | 14.4 |
| **1012** | O | Y | 16.13 | 13.6 | 300 | 15.5 | 96.1 | -3.9 | 11.7 | 30 | 0.02 | 9.7 |
| **1013** | O | Y | 7.0 | 28.6 | 190 | 7.7 | 110.0 | 10.0 | 30.7 | 90 | 0.1 | 32.3 |
| **1015** | O | Y | 12.12 | 15.2 | 180 | 12.17 | 100.4 | 0.4 | 16.3 | 100 | 0.02 | 11.6 |
| **1018** | O | Y | 1.67 | - | 90 | 1.77 | 106.0 | 6.0 | 6.9 | 90 | 0.05 | 10.2 |
| **1020** | O | Y | 6.23 | 13.2 | 270 | 3.3 | 53.0 | -47.0 | 10.8 | 0 | 0.01 | 14.8 |
| **1022** | O | Y | 15.8 | 8.9 | 90 | 21.8 | 138.0 | 38.0 | 7.2 | 90 | 0.1 | 10.8 |
| **1023** | O | Y | 20.4 | 17 | 180 | 21.5 | 105.4 | 5.4 | 14.4 | 200 | 0.17 | 12 |
| **1002** | OD | Y | 7.32 | 8.1 | 270 | - | - | - | 0.5 | 100 | 0.02 | 0.5 |
| **1004** | OD | N | 5.82 | 16.6 | - | - | - | - | - | - | 0.02 | 0.5 |
| **1005** | OD | Y | 24.0 | 13.0 | 225 | 11.7 | 48.8 | -51.3 | 0.9 | 180 | 0.1 | 1 |
| **1009** | OD | N | 24.55 | 24.9 | 300 | - | - | - | - | 270 | - | - |
| **1010** | OD | Y | 6.23 | 33.3 | 300 | 2.39 | 38.4 | -61.6 | 0.4 | 100 | 0.01 | 0.4 |
| **1014** | OD | Y | 3.59 | 12.7 | 270 | 1.46 | 40.7 | -59.3 | 0.6 | 100 | 0.02 | 0.6 |
| **1016** | OD | Y | 6.1 | 20.1 | 180 | 1.06 | 17.4 | -82.6 | 0.1 | 200 | 0.01 | 0.5 |
| **1017** | OD | Y | 20.26 | 5.9 | 270 | 13.42 | 66.2 | -33.8 | 0.4 | 200 | 0.01 | 0.4 |
| **1019** | OD | Y | 8.05 | 15.7 | 180 | 4.17 | 51.8 | -48.2 | 0.4 | 30 | 0.01 | 0.4 |
| **1021** | OD | Y | 8.61 | 17.9 | 300 | 2.65 | 30.8 | -69.2 | 0.5 | 100 | 0.02 | 0.5 |
| **1024** | OD | Y | 3.67 | 13.5 | 90 | 2.1 | 57.2 | -42.8 | 0.6 | 100 | 0.01 | 0.5 |

**Supplementary Table 4:** Summary table of PSA, testosterone and PAR score for individual patients in both cohorts (O=olaparib; OD= olaparib+degarelix)

**Supplementary Table 5**: Summary of germline and somatic pathogenic variants identified across all patients (likely pathogenic and pathogenic as reported in ClinVar and InterVar)

| **ID** | **Germline variants** | **VAF (%)** | **Classification** | **Somatic variants** | **VAF (%)** | **Classification** | **Structural variations** |
| --- | --- | --- | --- | --- | --- | --- | --- |
| **1001** | None | - | - | MET Q1029X  NF2 R262X | 6.5  5.6 | Pathogenic  Pathogenic | None |
| **1002** | None | - | - | PIK3CA E542A  PIK3CD E1021K | 2.7  4.3 | Pathogenic  Pathogenic | None |
| **1005** | None | - | - | JAK2 E596K  PIK3CD G781R  PIK3R2 T366M | 5.6  7.2  5.2 | Likely pathogenic  Likely pathogenic  Likely pathogenic | None |
| **1007** | None | - | - | None | - | - | None |
| **1008** | None | - | - | CHEK2 T519M  FLCN R527X  NOTCH1 R1984X | 4.8  3.6  2.3 | Likely pathogenic  Pathogenic  Pathogenic | None |
| **1010** | BRCA2 S1982fs | 48.3 | Pathogenic | ATM R250X  ATM R1730X  SPOP F133C | 4.3  3.4  4.3 | Pathogenic  Pathogenic  Likely pathogenic | None |
| **1011** | None | - | - | DNMT3B R840Q  TP63 R379C | 6.9  16.6 | Pathogenic  Likely pathogenic | None |
| **1012** | None | - | - | CTNNB1 D32Y  CTNNB1 S37C | 4.6  27.4 | Pathogenic  Pathogenic | None |
| **1014** | None | - | - | None | - | - | None |
| **1015** | None | - | - | EGFR R222C  MET R547X  SPOP F102V | 5.3  5.3  12.3 | Likely pathogenic  Pathogenic  Likely pathogenic | None |
| **1016** | None | - | - | None | - | - | None |
| **1017** | RAD54L R202C | 46.70 | Likely pathogenic |  |  |  | None |
| **1018** | None | - | - | AR Q80fs  TP53 V274A | 5.40  33.5 | Likely pathogenic  Likely pathogenic | None |
| **1019** | None | - | - | ATM R1875X  HNF1A G288fs  IDH1 R132C  MYC P72S | 3.6  7.0  14.1  2.6 | Pathogenic  Pathogenic  Pathogenic  Pathogenic | None |
| **1020** | None | - | - | PTEN R406X  TP63 R319H | 7.0  5.0 | Pathogenic  Pathogenic | None |
| **ID** | **Germline variants** | **VAF (%)** | **Classification** | **Somatic variants** | **VAF (%)** | **Classification** | **Structural variations** |
| **1021** | None | - | - | PIK3CA N345K | 21.5 | Pathogenic | PARP1P1 duplication (25%)  TMPRSS2 transversion (29%) |
| **1022** | None | - | - | ARID1A I692fs  ERCC2 R601Q  FOXA1 S250_G257del | 30.2  5.2  20 | Likely pathogenic  Pathogenic  Likely pathogenic | ETV6 deletion (43%)  PARP1P1 duplication (51%)  PARP1P1-PARP1 fusion (29%) |
| **1023** | None | - | - | AR R618W  ARAF G449S | 6.9  6 | Likely PathogenicLikely pathogenic | TMPRSS2-ERGfusion (39%) |
| **1024** |  |  |  | ABL1 R476H  FANCA Q1392X  MUTYH R19X  RB1 R251X | 5.5  7.2  3.8  5.2 | Likely pathogenic  Likely pathogenic  Pathogenic  Pathogenic | TMPRSS2-ERG fusion (28%) |

**
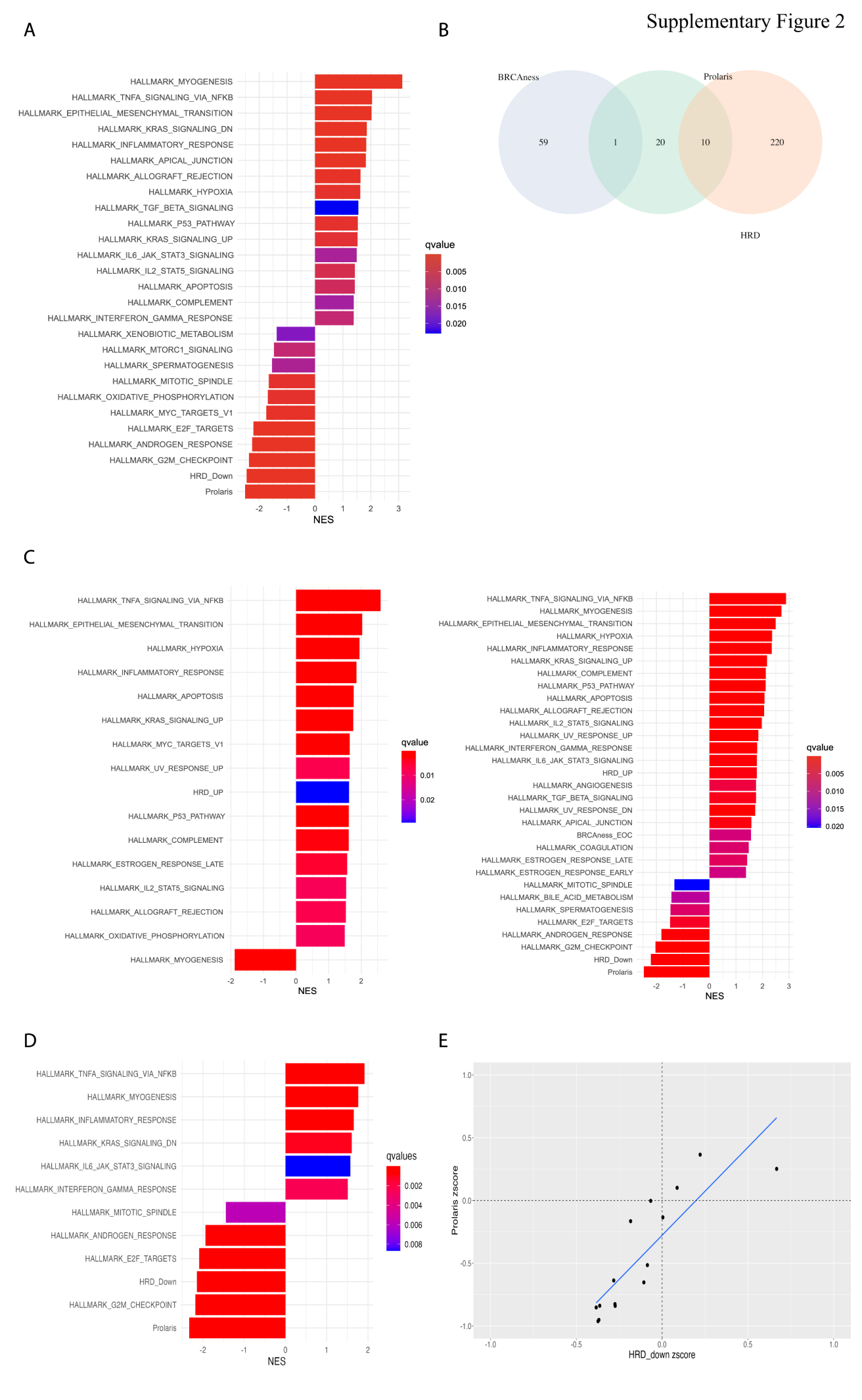
**

**Supplementary Figure 2:** Additional RNA sequencing analysis performed from CANCAP03 study. Gene set enrichment analysis of differential pathway enrichment between olaparib+degarelix and cohorts (B) Overlap between specific signatures which were used in gene set enrichment analysis in conjunction with the MSigDB Hallmark signatures. Prolaris from^43^, BRCAness from^44^, HRD from^45^. (C) Gene set enrichment analysis of patients excluding patient 1010 and 1019 harbouring HR deficiencies. (D) Gene set enrichment analysis of patients within the DARANA trial. Genes were ranked by Log2Fold change, with respect to pre-treatment control samples. Enrichment analysis was performed using the MSigDB cancer hallmarks and expression signatures relating to homologous recombination deficiency. (E) Correlation between Prolaris and HRD_down expression changes from patients in the DARANA study^37^.

**
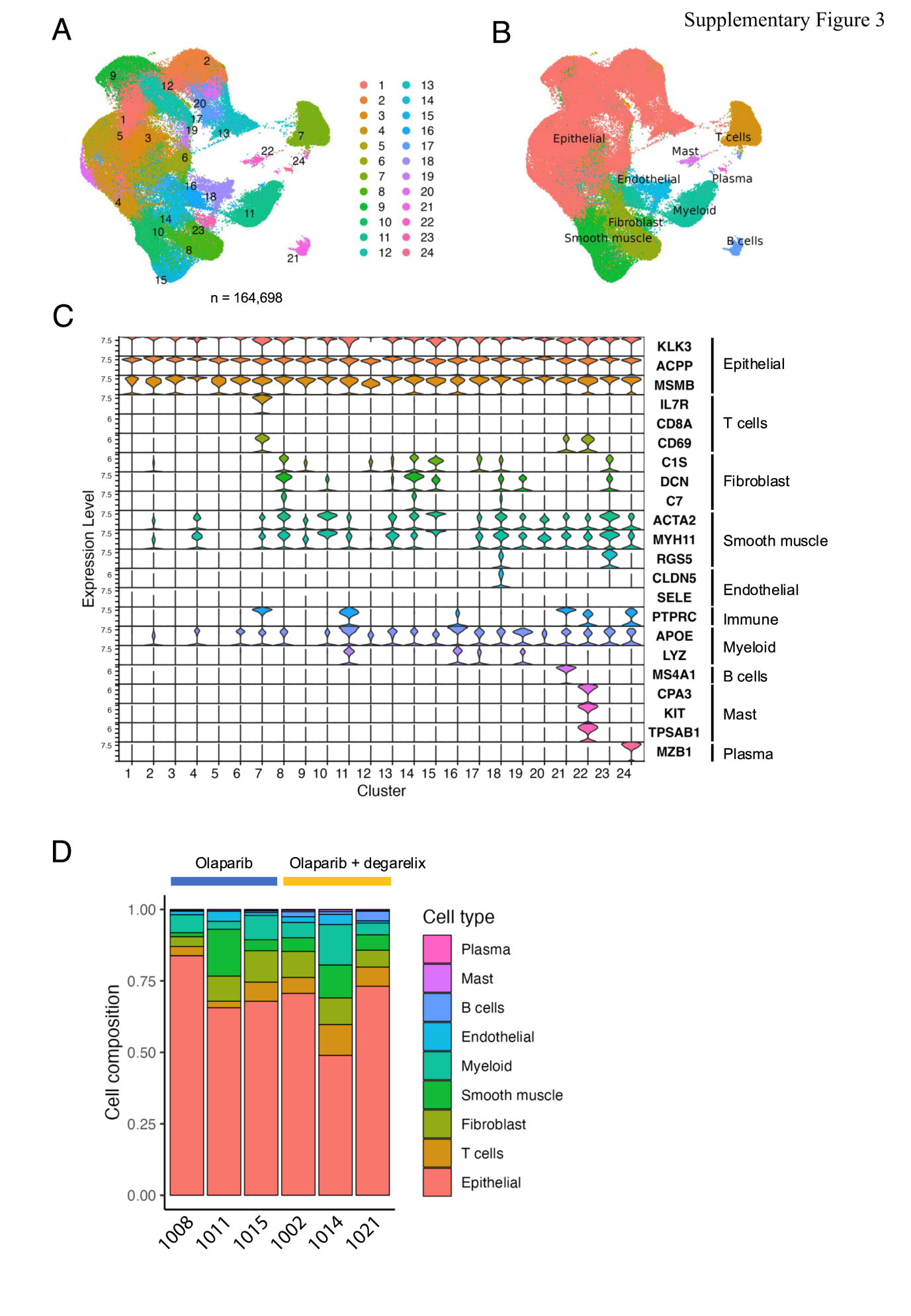
**

**Supplementary Figure 3:** Additional analysis of single-nuclear RNA-sequencing data. (A) UMAP of single-nuclear RNA-seq clusters. (B) UMAP showing major cell types identified. (C) Violin plot of expression of major prostate cancer cell type markers^35^ across clusters, used for cell type annotations (D) Distribution of major cell types in samples across olaparib and olaparib + degarelix treatment conditions.

| Cancer type(s)/subtype(s)/ stage(s)/condition | Prostate Cancer/ Localised (untreated)/ Stage 2-3B |
| --- | --- |
| Considerations related to: | |
| Sex | Prostate Cancer affects men only and is the second leading cause of cancer related death in men. |
| Age | About 1 in 8 men will be diagnosed with prostate cancer during their lifetime. But each man’s risk of prostate cancer can vary, based on his age, race/ethnicity, and other factors.  For example, prostate cancer is more likely to develop in older men. About 6 in 10 prostate cancers are diagnosed in men who are 65 or older, and it is rare in men under 40. The average age of men when they are first diagnosed is about 67. |
| Race/ethnicity | An estimate of the racial/ethnic distribution of Prostate Cancer in the USA was reported as follows: white 69.3%, Black 12.0%, Hispanic 10.5%, Asian and Pacific Islanders 7.7%, and American Indian or Alaskan native patients 0.5% (Zeng et al, Lancet 2023).  Prostate cancer risk is also higher in African American men and in Caribbean men of African ancestry than in men of other races. |
| Geography | The American Cancer Society’s estimates for prostate cancer in the United States for 2024 are: About 299,010 new cases of prostate cancer and about 35,250 deaths from prostate cancer |
| Other considerations | Men who undergo radical prostatectomy are expected to be younger, with fewer medical comorbidities than the average population of men with Prostate Cancer  The number of prostate cancers diagnosed each year declined sharply from 2007 to 2014, coinciding with fewer men being screened because of changes in screening recommendations. Since 2014, however, the incidence rate has increased by 3% per year overall and by about 5% per year for advanced-stage prostate cancer. |
| Overall representativeness of this study | Although our study only recruited a small number of men, they are representative of those undergoing radical prostatectomy in the United Kingdom, with an age range between 47-71 years.  Participants recruited to our study were predominantly white, again representative of the local population. Future studies should aim to widen participation and include patients from other ethnicities. |

**Supplementary Table 6.** Representativeness of Study Participants
